# Supplementary material for: Multi-step vs. single-step resistance evolution under different drugs, pharmacokinetics, and treatment regimens
Source: eLife. 2021 May 18;10:e64116. doi: 10.7554/eLife.64116 (PMC8184216; doi:10.7554/eLife.64116)
Supplement: Supplementary file 1. — Parameter values and units used in the pharmacodynamic model for either antibiotic or antimicrobial peptide simulations are shown. [file elife-64116-supp1.docx]

| **Parameters** | **Value** | **Unit** | **Description** |
| --- | --- | --- | --- |
| *r* | 1 | h^-1^ | Replication rate |
| *b* | 2-100 | xMIC | Benefit per mutation |
| *c* | 0.008-0.2 | - | Cost per mutation |
| *u* | 3*10^-6^, 1*10^-6^ | Probability per cell division | Mutation rate (AB, AMP) |
| *K* | 10^9^ | CFU | Carrying capacity |
| $\gamma$ | 0.01 | h^-1^ | Death rate |
| $\psi_{min}$ | -5, -50 | h^-1^ | Min. growth rate (AB, AMP) |
| *a* | 0.1-100 | xMIC | Drug concentration |
| *k_a_* | 0.5 | h^-1^ | Drug absorption rate |
| *k* | 0.1 | h^-1^ | Drug decay rate |
| *κ* | 1.5, 5 | - | Steepness of the PD curve (AB, AMP) |
| *τ* | 1/24 | h^-1^ | Dose frequency |
| *k2cmax* | 12-96 | h | Time till max. dose |
| *M_i_* | 0-10^9^ | CFU | Population with i mutations |
| *M_pi_* | 0-10^9^ | CFU | Population with a plasmid and i mutations |
| *b_p_* | 0.6-24 | xMIC | Benefit of the plasmid |
| *c_p_* | 0.03-0.1 | - | Cost of the plasmid |
| *α* | 3*10^-10^ | h^-1^ | Plasmid acquisition rate from the environment |
| *β* | 3*10^-8^ | h^-1^ | Plasmid acquisition rate from *M_pi_* carriers |
| $P_{max}$ | 10^5^ | CFU | Max. acceptable pathogen burden |
